# Supplementary material for: Effects of a Web-Based Lifestyle Intervention on Physical Fitness and Health in Physically Inactive Adults: A Randomized Controlled Trial
Source: Healthcare (Basel). 2023 Oct 29;11(21):2847. doi: 10.3390/healthcare11212847 (PMC10649962; doi:10.3390/healthcare11212847)
Supplement: Supplementary file 1 [file healthcare-11-02847-s001.zip › healthcare-2665996-supplementary.pdf]

Table S1. Descriptive statistics for physical activity behavior (Fitbit Charge 3™) (ITT analysis, n = 189).

Group	t0 (0 months)	t1 (3 months)	t2 (9 months)	t3 (15 months)
Daily step count (steps/day)				
Intervention	10,298.98 ± 3275.36	10,408.19 ± 2926.62	10,755.86 ± 2884.27	10,342.08 ± 2726.89
Control	10,110.05 ± 2934.87	10,380.37 ± 3345.13	10,278.82 ± 3091.26	10,425.40 ± 3059.40
Sedentary behavior (min/day)				
Intervention	849.96 ± 203.63	807.93 ± 207.69	802.23 ± 178.30	831.00 ± 178.49
Control	820.93 ± 172.93	808.71 ± 225.12	825.55 ± 176.66	792.49 ± 164.76
Light intensity physical activity (min/week)				
Intervention	1928.94 ± 529.11	1976.09 ± 461.52	1964.52 ± 463.49	1907.21 ± 443.50
Control	1857.32 ± 394.10	1959.54 ± 413.05	1862.30 ± 370.63	1886.68 ± 332.07
Moderate intensity physical activity (min/week)				
Intervention	162.93 ± 135.31	152.52 ± 76.30	157.70 ± 81.57	169.98 ± 91.68
Control	174.50 ± 108.09	158.67 ± 86.47	171.39 ± 166.54	178.41 ± 121.56
Vigorous intensity physical activity (min/week)				
Intervention	179.44 ± 115.91	173.50 ± 85.94	190.22 ± 102.79	187.45 ± 71.59
Control	204.04 ± 136.16	192.11 ± 151.15	207.65 ± 154.89	194.84 ± 129.13
Total physical activity (min/week)				
Intervention	2276.06 ± 572.77	2315.62 ± 503.16	2315.72 ± 519.43	2259.61 ± 511.09
Control	2229.46 ± 423.58	2311.92 ± 480.27	2247.68 ± 488.40	2257.68 ± 423.92

Data are presented as mean ± SD. Intervention: n = 104, control: n = 85. ITT: intention-to-treat.

Table S2. Effect sizes for physical activity behavior (Fitbit Charge 3^{TM}) (ITT analysis, *n* = 189).

**Group**	**t0-t1 (0-3 months)**	**t0-t2 (0-9 months)**	**t0-t3 (0-15 months)**
Daily step count (steps/day)			
Intervention	0.04 [-0.24, 0.31]	0.15 [-0.12, 0.42]	0.01 [-0.26, 0.29]
Control	0.09 [-0.22, 0.39]	0.06 [-0.25, 0.36]	0.11 [-0.20, 0.41]
Sedentary behavior (min/day)			
Intervention	-0.20 [-0.48, 0.07]	-0.25 [-0.52, 0.02]	-0.10 [-0.37, 0.17]
Control	-0.06 [-0.36, 0.24]	0.03 [-0.27, 0.33]	-0.17 [-0.47, 0.13]
Light intensity physical activity (min/week)			
Intervention	0.10 [-0.18, 0.37]	0.07 [-0.20, 0.34]	-0.05 [-0.32, 0.23]
Control	0.25 [-0.05, 0.56]	0.01 [-0.29, 0.31]	0.08 [-0.22, 0.38]
Moderate intensity physical activity (min/week)			
Intervention	-0.10 [-0.37, 0.18]	-0.05 [-0.32, 0.23]	0.06 [-0.21, 0.33]
Control	-0.16 [-0.46, 0.14]	-0.02 [-0.32, 0.28]	0.03 [-0.77, 0.34]
Vigorous intensity physical activity (min/week)			
Intervention	-0.06 [-0.33, 0.21]	0.10 [-0.17, 0.37]	0.08 [-0.19, 0.36]
Control	-0.08 [-0.38, 0.22]	0.03 [-0.28, 0.33]	-0.07 [-0.37, 0.23]
Total physical activity (min/week)			
Intervention	0.07 [-0.20, 0.35]	0.07 [-0.20, 0.34]	-0.03 [-0.30, 0.24]
Control	0.18 [-0.12, 0.48]	0.04 [-0.26, 0.34]	0.07 [-0.23, 0.37]

Data are presented as Cohen's *d* [± 95% CI]. Interpretation: |*d*| = 0.2: small effect, |*d*| = 0.5: medium effect, |*d*| = 0.8: large effect. Intervention: *n* = 104, control: *n* = 85. ITT: intention-to-treat.

Table S3. Results of robust linear mixed models for physical activity behavior (Fitbit Charge 3™) (ITT analysis, $n = 189$).

**Predictors**	**Daily step count (steps/day)**		**Sedentary behavior (min/day)**		**Light intensity physical activity (min/week)**		**Moderate intensity physical activity (min/week)**		**Vigorous intensity physical activity (min/week)**		**Total physical activity (min/week)**	
	Estimate	*p*-value	Estimate	*p*-value	Estimate	*p*-value	Estimate	*p*-value	Estimate	*p*-value	Estimate	*p*-value
(Intercept)	10,308.11 ± 703.31	< 0.001*	873.06 ± 51.00	< 0.001*	1950.97 ± 111.57	< 0.001*	129.53 ± 20.01	< 0.001*	150.04 ± 23.94	< 0.001*	2268.19 ± 120.41	< 0.001*
Time												
t0-t1 (0-3 months)	51.16 ± 846.64	0.952	-85.80 ± 62.18	0.168	3.28 ± 133.31	0.980	5.85 ± 26.87	0.828	14.99 ± 31.46	0.634	2.27 ± 160.99	0.989
t0-t2 (0-9 months)	809.27 ± 925.04	0.382	-110.33 ± 61.43	0.073	64.32 ± 145.07	0.658	14.65 ± 31.63	0.644	19.69 ± 33.71	0.559	82.87 ± 149.24	0.579
t0-t3 (0-15 months)	-19.38 ± 856.73	0.982	-12.76 ± 61.34	0.835	-34.85 ± 134.49	0.796	23.36 ± 27.07	0.388	30.66 ± 32.31	0.343	-6.16 ± 139.32	0.965
Group (control)	-200.82 ± 459.09	0.662	-29.19 ± 33.44	0.383	-59.06 ± 72.66	0.417	14.70 ± 13.14	0.264	17.50 ± 15.66	0.264	-29.90 ± 78.20	0.702
Time*group (control)												
t0-t1 (0-3 months)	92.37 ± 555.99	0.868	35.24 ± 40.09	0.380	56.91 ± 86.31	0.510	-9.11 ± 17.55	0.604	-16.19 ± 20.46	0.429	40.28 ± 100.47	0.689
t0-t2 (0-9 months)	-344.20 ± 604.98	0.570	57.54 ± 39.79	0.149	-21.95 ± 88.75	0.805	-11.71 ± 20.68	0.572	-12.71 ± 22.11	0.566	-35.47 ± 94.96	0.709
t0-t3 (0-15 months)	124.00 ± 566.13	0.827	-9.26 ± 40.49	0.819	34.57 ± 80.29	0.667	-14.62 ± 18.50	0.430	-20.08 ± 21.07	0.341	16.17 ± 91.33	0.860

Data are presented as regression coefficient ± SE. Statistical significance ($p < 0.05$) is indicated (*). Intervention: $n = 104$, control: $n = 85$. ITT: intention-to-treat.

Table S4. Descriptive statistics for physical activity behavior (IPAQ-L) (ITT analysis, n = 189).

**Group**	**t0 (0 months)**	**t1 (3 months)**	**t2 (9 months)**	**t3 (15 months)**
Sedentary behavior (min/day)				
Intervention	443.36 ± 167.62	439.58 ± 147.93	449.02 ± 109.20	458.46 ± 120.93
Control	471.44 ± 169.04	437.08 ± 163.50	465.31 ± 144.62	488.96 ± 128.98
Light intensity physical activity (min/week)				
Intervention	325.02 ± 537.38	390.68 ± 442.65	381.71 ± 315.26	320.47 ± 326.88
Control	253.89 ± 372.51	346.01 ± 400.61	344.68 ± 338.39	283.21 ± 190.10
Moderate intensity physical activity (min/week)				
Intervention	434.58 ± 483.85	651.80 ± 627.46	613.69 ± 500.82	569.41 ± 412.08
Control	421.54 ± 481.48	666.34 ± 765.83	562.33 ± 511.38	563.56 ± 489.46
Vigorous intensity physical activity (min/week)				
Intervention	25.74 ± 129.73	127.80 ± 248.09	131.73 ± 294.81	77.23 ± 104.61
Control	35.94 ± 147.92	120.28 ± 240.23	102.65 ± 218.47	62.15 ± 71.49
Total physical activity (min/week)				
Intervention	785.34 ± 823.80	1196.30 ± 957.27	1121.94 ± 808.06	1020.21 ± 620.47
Control	711.38 ± 771.52	1145.70 ± 1049.06	986.51 ± 799.02	931.66 ± 594.69

Data are presented as mean ± SD. Intervention: n = 104, control: n = 85. ITT: intention-to-treat; IPAQ-L: International Physical Activity Questionnaire (long version).

Table S5. Effect sizes for physical activity behavior (IPAQ-L) (ITT analysis, *n* = 189).

**Group**	**t0-t1 (0-3 months)**	**t0-t2 (0-9 months)**	**t0-t3 (0-15 months)**
Sedentary behavior (min/day)			
Intervention	-0.02 [-0.30, 0.25]	0.04 [-0.23, 0.31]	0.10 [-0.17, 0.37]
Control	-0.21 [-0.51, 0.10]	-0.04 [-0.34, 0.26]	0.12 [-0.19, 0.412]
Light intensity physical activity (min/week)			
Intervention	0.13 [-0.14, 0.41]	0.13 [-0.14, 0.40]	-0.01 [-0.28, 0.26]
Control	0.24 [-0.06, 0.54]	0.26 [-0.05, 0.56]	0.10 [-0.20, 0.40]
Moderate intensity physical activity (min/week)			
Intervention	0.39 [0.11, 0.66]	0.36 [0.09, 0.64]	0.30 [0.03, 0.57]
Control	0.38 [0.08, 0.69]	0.28 [-0.02, 0.59]	0.29 [-0.01, 0.59]
Vigorous intensity physical activity (min/week)			
Intervention	0.52 [0.24, 0.79]	0.47 [0.19, 0.74]	0.44 [0.16, 0.71]
Control	0.42 [0.12, 0.73]	0.36 [0.05, 0.66]	0.23 [-0.08, 0.53]
Total physical activity (min/week)			
Intervention	0.46 [0.18, 0.74]	0.41 [0.14, 0.69]	0.32 [0.05, 0.60]
Control	0.47 [0.17, 0.78]	0.35 [0.05, 0.65]	0.32 [0.02, 0.62]

Data are presented as Cohen's *d* [± 95% CI]. Interpretation: |*d*| = 0.2: small effect, |*d*| = 0.5: medium effect, |*d*| = 0.8: large effect. Intervention: *n* = 104, control: *n* = 85. ITT: intention-to-treat; IPAQ-L: International Physical Activity Questionnaire (long version).

Table S6. Results of robust linear mixed models for physical activity behavior (IPAQ-L) (ITT analysis, n = 189).

Predictors	Sedentary behavior (min/day)		Light intensity physical activity (min/week)		Moderate intensity physical activity (min/week)		Vigorous intensity physical activity (min/week)		Total physical activity (min/week)	
	Estimate	*p*-value	Estimate	*p*-value	Estimate	*p*-value	Estimate	*p*-value	Estimate	*p*-value
(Intercept)	405.51 ± 35.71	< 0.001*	234.66 ± 49.37	< 0.001*	371.48 ± 85.27	< 0.001*	2.06 ± 13.92	0.882	692.17 ± 137.29	< 0.001*
Time										
t0-t1 (0-3 months)	33.22 ± 39.85	0.405	66.83 ± 68.19	0.328	142.20 ± 105.37	0.178	81.86 ± 20.04	< 0.001*	288.73 ± 176.23	0.102
t0-t2 (0-9 months)	26.59 ± 40.89	0.516	111.71 ± 66.76	0.095	184.21 ± 104.97	0.080	59.76 ± 22.37	0.008*	335.26 ± 194.05	0.085
t0-t3 (0-15 months)	21.41 ± 38.84	0.582	-2.55 ± 70.86	0.971	100.95 ± 113.47	0.374	40.16 ± 20.98	0.056	186.58 ± 183.27	0.309
Group (control)	31.86 ± 23.26	0.171	-20.11 ± 32.20	0.532	-19.15 ± 55.71	0.731	7.85 ± 9.08	0.387	-50.72 ± 89.08	0.569
Time*group (control)										
t0-t1 (0-3 months)	-32.63 ± 25.85	0.207	6.13 ± 43.81	0.889	-0.01 ± 68.62	1.000	-23.19 ± 13.49	0.086	26.91 ± 112.08	0.810
t0-t2 (0-9 months)	-16.90 ± 28.42	0.552	-25.35 ± 42.55	0.551	-41.97 ± 67.48	0.534	-16.95 ± 14.21	0.233	-49.85 ± 124.87	0.690
t0-t3 (0-15 months)	0.062 ± 25.71	0.998	27.31 ± 44.17	0.537	-0.31 ± 71.43	0.996	-8.23 ± 13.61	0.546	10.97 ± 122.20	0.929

Data are presented as regression coefficient ± SE. Statistical significance (p < 0.05) is indicated (*). Intervention: n = 104, control: n = 85. ITT: intention-to-treat; IPAQ-L: International Physical Activity Questionnaire (long version).

Table S7. Descriptive statistics for dietary behavior (ITT analysis, n = 189).

**Group**	**t0 (0 months)**	**t1 (3 months)**	**t2 (9 months)**	**t3 (15 months)**
Energy intake (kcal/day)				
Intervention	1941.23 ± 599.36	1833.72 ± 503.54	1887.85 ± 421.60	1859.62 ± 364.42
Control	2001.47 ± 548.75	1887.95 ± 479.22	1859.15 ± 390.06	1857.71 ± 378.19
Protein intake (g/day)				
Intervention	71.58 ± 20.93	63.99 ± 16.29	71.62 ± 16.49	68.53 ± 13.96
Control	70.63 ± 20.51	66.13 ± 16.57	66.96 ± 16.78	69.55 ± 15.66
Fat intake (g/day)				
Intervention	75.95 ± 30.60	71.11 ± 24.24	75.05 ± 18.80	73.81 ± 20.61
Control	79.74 ± 28.98	77.30 ± 26.21	75.84 ± 21.56	74.91 ± 19.05
Carbohydrate intake (g/day)				
Intervention	212.84 ± 71.64	205.46 ± 59.35	201.91 ± 53.18	202.24 ± 45.65
Control	217.65 ± 65.82	204.14 ± 54.34	196.03 ± 42.35	201.82 ± 47.97
Alcohol intake (g/day)				
Intervention	9.30 ± 13.84	8.86 ± 13.20	6.86 ± 10.63	10.47 ± 10.32
Control	9.42 ± 11.79	8.79 ± 11.93	8.34 ± 8.12	7.70 ± 6.41
Fiber intake (g/day)				
Intervention	22.73 ± 8.88	20.09 ± 6.22	20.21 ± 5.36	20.27 ± 5.17
Control	22.29 ± 8.75	20.61 ± 6.73	20.32 ± 6.57	21.10 ± 6.07
HEI-NVS (score)				
Intervention	77.53 ± 8.92	75.49 ± 8.80	75.80 ± 8.10	75.14 ± 8.18
Control	76.59 ± 9.53	75.84 ± 8.43	75.67 ± 8.94	76.83 ± 7.22

Data are presented as mean ± SD. Intervention: n = 104, control: n = 85. ITT: intention-to-treat; HEI-NVS: Healthy Eating Index of the German National Nutrition Survey.

Table S8. Effect sizes for dietary behavior (ITT analysis, n = 189).

Group	t0-t1 (0-3 months)	t0-t2 (0-9 months)	t0-t3 (0-15 months)
Energy intake (kcal/day)			
Intervention	-0.19 [-0.47, 0.08]	-0.10 [-0.38, 0.17]	-0.17 [-0.44, 0.11]
Control	-0.22 [-0.52, 0.08]	-0.30 [-0.60, 0.00]	-0.31 [-0.61, -0.00]
Protein intake (g/day)			
Intervention	-0.41 [-0.68, -0.13]	0.00 [-0.27, 0.27]	-0.17 [-0.44, 0.10]
Control	-0.24 [-0.54, 0.06]	-0.20 [-0.50, 0.11]	-0.06 [-0.36, 0.24]
Fat intake (g/day)			
Intervention	-0.18 [-0.45, 0.10]	-0.04 [-0.31, 0.24]	-0.08 [-0.35, 0.19]
Control	-0.09 [-0.39, 0.21]	-0.15 [-0.45, 0.15]	-0.12 [-0.50, 0.11]
Carbohydrate intake (g/day)			
Intervention	-0.11 [-0.38, 0.16]	-0.17 [-0.45, 0.10]	-0.18 [-0.45, 0.10]
Control	-0.22 [-0.53, 0.08]	-0.39 [-0.69, -0.09]	-0.28 [-0.58, 0.03]
Alcohol intake (g/day)			
Intervention	-0.03 [-0.31, 0.24]	-0.20 [-0.47, 0.08]	0.10 [-0.18, 0.37]
Control	-0.05 [-0.35, 0.25]	-0.11 [-0.41, 0.20]	-0.18 [-0.48, 0.12]
Fiber intake (g/day)			
Intervention	-0.34 [-0.62, -0.07]	-0.34 [-0.62, -0.07]	-0.34 [-0.61, -0.06]
Control	-0.22 [-0.52, 0.09]	-0.26 [-0.56, 0.05]	-0.16 [-0.46, 0.14]
HEI-NVS (score)			
Intervention	-0.23 [-0.50, 0.04]	-0.20 [-0.48, 0.07]	-0.28 [-0.55, -0.01]
Control	-0.08 [-0.38, 0.22]	-0.10 [-0.40, 0.20]	0.03 [-0.27, 0.33]

Data are presented as Cohen's d [± 95% CI]. Interpretation: $|d|$ = 0.2: small effect, $|d|$ = 0.5: medium effect, $|d|$ = 0.8: large effect. Intervention: n = 104, control: n = 85. ITT: intention-to-treat; HEI-NVS: Healthy Eating Index of the German National Nutrition Survey.

Table S9. Results of robust linear mixed models for dietary behavior (ITT analysis, *n* = 189).

Predictors	Energy intake (kcal/day)		Protein intake (g/day)		Fat intake (g/day)		Carbohydrate intake (g/day)		Alcohol intake (g/day)		Fiber intake (g/day)		HEI-NVS (score)	
	Estimate	*p*-value	Estimate	*p*-value	Estimate	*p*-value	Estimate	*p*-value	Estimate	*p*-value	Estimate	*p*-value	Estimate	*p*-value
(Intercept)	1790.72 ± 115.87	< 0.001*	70.91 ± 3.99	< 0.001*	66.32 ± 5.73	< 0.001*	203.80 ± 13.69	< 0.001*	6.43 ± 1.54	< 0.001*	22.32 ± 1.60	< 0.001*	79.21 ± 2.07	< 0.001*
Time														
t0-t1 (0-3 months)	-30.55 ± 137.24	0.824	-9.27 ± 4.94	0.061	-2.72 ± 7.04	0.700	0.23 ± 15.81	0.988	-1.19 ± 1.74	0.493	-2.93 ± 1.84	0.112	-3.33 ± 2.63	0.207
t0-t2 (0-9 months)	92.21 ± 143.62	0.521	2.93 ± 5.48	0.593	6.58 ± 7.00	0.349	0.18 ± 15.62	0.991	-3.74 ± 1.81	0.039*	-2.23 ± 1.90	0.241	-2.50 ± 2.61	0.338
t0-t3 (0-15 months)	41.34 ± 156.02	0.791	-5.13 ± 6.05	0.397	4.34 ± 8.26	0.600	0.31 ± 17.17	0.986	2.15 ± 2.12	0.312	-3.17 ± 1.95	0.104	-4.51 ± 2.74	0.100
Group (control)	103.24 ± 75.50	0.172	-0.61 ± 2.61	0.813	6.01 ± 3.75	0.110	5.80 ± 8.99	0.519	0.43 ± 1.01	0.666	-0.49 ± 1.04	0.641	-1.27 ± 1.35	0.346
Time*group (control)														
t0-t1 (0-3 months)	-53.19 ± 89.92	0.554	2.18 ± 3.22	0.498	-0.17 ± 4.55	0.971	-7.28 ± 10.16	0.474	0.20 ± 1.13	0.857	0.75 ± 1.19	0.531	1.29 ± 1.72	0.453
t0-t2 (0-9 months)	-121.47 ± 93.38	0.194	-3.23 ± 3.60	0.369	-5.25 ± 4.63	0.257	-10.35 ± 10.18	0.310	1.51 ± 1.21	0.212	0.28 ± 1.22	0.815	0.87 ± 1.70	0.609
t0-t3 (0-15 months)	-101.72 ± 100.14	0.310	1.82 ± 3.73	0.625	-4.84 ± 5.52	0.382	-9.46 ± 10.85	0.384	-1.70 ± 1.32	0.200	1.18 ± 1.27	0.353	2.30 ± 1.72	0.181

Data are presented as regression coefficient ± SE. Statistical significance ($p < 0.05$) is indicated (*). Intervention: *n* = 104, control: *n* = 85. ITT: intention-to-treat; HEI-NVS: Healthy Eating Index of the German National Nutrition Survey.

Table S10. Descriptive statistics for anthropometry and blood pressure (ITT analysis, *n* = 189).

Group	t0<br>(0 months)	t1<br>(3 months)	t2<br>(9 months)	t3<br>(15 months)
Body weight (kg)				
Intervention	74.99 ± 13.01	73.96 ± 11.81	74.35 ± 10.59	73.70 ± 10.09
Control	74.25 ± 14.57	73.31 ± 12.81	73.85 ± 12.04	73.95 ± 11.49
Body height (m)				
Intervention	1.70 ± 0.08	1.70 ± 0.08	1.70 ± 0.07	1.70 ± 0.07
Control	1.71 ± 0.08	1.71 ± 0.07	1.71 ± 0.07	1.71 ± 0.06
BMI (kg/m^2)				
Intervention	25.86 ± 3.50	25.54 ± 3.04	25.27 ± 2.60	25.32 ± 2.69
Control	25.19 ± 3.84	24.95 ± 3.42	25.46 ± 3.42	25.35 ± 3.23
Fat mass (kg)				
Intervention	25.56 ± 7.78	25.01 ± 6.72	24.88 ± 5.35	24.44 ± 5.79
Control	24.68 ± 7.28	24.34 ± 6.68	24.65 ± 6.72	24.41 ± 6.23
Fat-free mass (kg)				
Intervention	49.43 ± 9.76	48.58 ± 9.03	48.88 ± 8.09	48.60 ± 7.67
Control	49.18 ± 10.79	48.96 ± 9.60	48.69 ± 8.41	49.13 ± 8.12
Waist circumference (cm)				
Intervention	89.29 ± 10.89	88.06 ± 9.14	87.85 ± 7.78	86.67 ± 8.13
Control	87.32 ± 12.06	86.77 ± 9.68	86.88 ± 9.54	87.02 ± 9.13
Systolic blood pressure (mmHg)				
Intervention	123.29 ± 12.58	124.32 ± 14.03	125.17 ± 11.77	126.57 ± 11.98
Control	125.82 ± 14.24	124.82 ± 15.13	124.47 ± 11.64	126.25 ± 11.52
Diastolic blood pressure (mmHg)				
Intervention	83.04 ± 8.37	83.78 ± 8.62	84.83 ± 7.72	84.28 ± 6.95
Control	86.06 ± 9.22	85.21 ± 9.80	84.66 ± 7.22	84.98 ± 7.75
Heart rate (bpm)				
Intervention	66.24 ± 9.70	63.08 ± 8.56	64.66 ± 6.97	64.83 ± 7.01
Control	69.39 ± 11.68	65.88 ± 9.14	65.04 ± 7.49	65.04 ± 7.39

Data are presented as mean ± SD. Intervention: *n* = 104, control: *n* = 85. ITT: intention-to-treat; BMI: body mass index.

Table S11. Effect sizes for anthropometry and blood pressure (ITT analysis, n = 189).

Group	**t0-t1 (0-3 months)**	**t0-t2 (0-9 months)**	**t0-t3 (0-15 months)**
Body weight (kg)			
Intervention	-0.08 [-0.35, 0.19]	-0.05 [-0.33, 0.22]	-0.11 [-0.38, 0.16]
Control	-0.07 [-0.37, 0.23]	-0.03 [-0.33, 0.27]	-0.02 [-0.32, 0.28]
Body height (m)			
Intervention	0.00 [-0.27, 0.27]	-0.01 [-0.28, 0.26]	0.02 [-0.25, 0.29]
Control	-0.01 [-0.31, 0.29]	-0.04 [-0.34, 0.26]	0.02 [-0.28, 0.32]
BMI (kg/m^2)			
Intervention	-0.01 [-0.37, 0.178]	-0.19 [-0.46, 0.09]	-0.17 [-0.45, 0.10]
Control	-0.07 [-0.37, 0.24]	0.07 [-0.23, 0.38]	0.05 [-0.26, 0.35]
Fat mass (kg)			
Intervention	-0.08 [-0.35, 0.20]	-0.10 [-0.37, 0.17]	-0.16 [-0.44, 0.11]
Control	-0.05 [-0.35, 0.25]	-0.00 [-0.31, 0.30]	-0.04 [-0.34, 0.26]
Fat-free mass (kg)			
Intervention	-0.09 [-0.36, 0.18]	-0.06 [-0.33, 0.21]	-0.09 [-0.37, 0.18]
Control	-0.02 [-0.32, 0.28]	-0.05 [-0.35, 0.25]	-0.01 [-0.31, 0.30]
Waist circumference (cm)			
Intervention	-0.12 [-0.40, 0.15]	-0.15 [-0.43, 0.12]	-0.27 [-0.55, 0.00]
Control	-0.05 [-0.35, 0.25]	-0.04 [-0.34, 0.26]	-0.03 [-0.33, 0.27]
Systolic blood pressure (mmHg)			
Intervention	0.08 [-0.20, 0.35]	0.15 [-0.12, 0.43]	0.27 [-0.01, 0.54]
Control	-0.07 [-0.37, 0.23]	-0.10 [-0.41, 0.20]	0.03 [-0.27, 0.33]
Diastolic blood pressure (mmHg)			
Intervention	0.09 [-0.18, 0.36]	0.22 [-0.05, 0.50]	0.16 [-0.11, 0.43]
Control	-0.09 [-0.39, 0.21]	-0.17 [-0.47, 0.13]	-0.13 [-0.43, 0.17]
Heart rate (bpm)			
Intervention	-0.35 [-0.62, -0.07]	-0.19 [-0.46, 0.09]	-0.17 [-0.44, 0.11]
Control	-0.34 [-0.64, -0.03]	-0.44 [-0.75, -0.14]	-0.45 [-0.75, -0.14]

Data are presented as Cohen's d [± 95% CI]. Interpretation: $|d|$ = 0.2: small effect, $|d|$ = 0.5: medium effect, $|d|$ = 0.8: large effect. Intervention: n = 104, control: n = 85. ITT: intention-to-treat; BMI: body mass index.

Table S12. Results of robust linear mixed models for anthropometry and blood pressure (ITT analysis, n = 189).

Predictors	Body weight (kg)		Body height (m)		BMI (kg/m²)		Fat mass (kg)		Fat-free mass (kg)		Waist circumference (cm)		Systolic blood pressure (mmHg)		Diastolic blood pressure (mmHg)		Heart rate (bpm)	
	Estimate	*p*-value	Estimate	*p*-value	Estimate	*p*-value	Estimate	*p*-value	Estimate	*p*-value	Estimate	*p*-value	Estimate	*p*-value	Estimate	*p*-value	Estimate	*p*-value
(Intercept)	74.29 ± 2.83	< 0.001*	1.69 ± 0.02	< 0.001*	26.36 ± 0.76	< 0.001*	25.83 ± 1.52	< 0.001*	47.78 ± 1.82	< 0.001*	91.12 ± 2.31	< 0.001*	120.84 ± 3.08	< 0.001*	80.35 ± 1.92	< 0.001*	63.68 ± 2.10	< 0.001*
Time																		
t0-t1 (0-3 months)	-0.95 ± 1.62	0.558	-0.00 ± 0.01	0.898	-0.24 ± 0.49	0.632	-0.44 ± 1.05	0.675	-0.96 ± 0.91	0.292	-1.55 ± 1.71	0.367	3.07 ± 3.04	0.313	2.87 ± 1.85	0.121	-2.74 ± 2.15	0.203
t0-t2 (0-9 months)	-1.60 ± 2.17	0.462	-0.00 ± 0.01	0.917	-1.04 ± 0.68	0.132	-1.15 ± 1.17	0.329	-0.89 ± 0.85	0.297	-2.31 ± 1.92	0.231	4.76 ± 3.02	0.115	4.58 ± 2.20	0.039*	0.48 ± 2.69	0.860
t0-t3 (0-15 months)	-1.76 ± 1.96	0.372	-0.00 ± 0.01	0.919	-0.84 ± 0.67	0.217	-1.59 ± 1.10	0.148	-1.23 ± 1.06	0.249	-3.81 ± 1.84	0.039*	5.40 ± 3.03	0.075	3.96 ± 2.14	0.066	1.15 ± 2.24	0.607
Group (control)	-0.46 ± 1.85	0.802	0.01 ± 0.01	0.494	-0.70 ± 0.49	0.154	-0.78 ± 0.98	0.429	-0.20 ± 1.16	0.860	-2.23 ± 1.52	0.141	2.17 ± 2.01	0.279	2.85 ± 1.25	0.023*	2.69 ± 1.37	0.049*
Time*group (control)																		
t0-t1 (0-3 months)	0.18 ± 1.05	0.863	0.00 ± 0.00	0.884	0.04 ± 0.31	0.885	-0.02 ± 0.67	0.982	0.53 ± 0.58	0.366	0.55 ± 1.12	0.623	-2.47 ± 2.01	0.220	-2.07 ± 1.20	0.086	-0.47 ± 1.40	0.737
t0-t2 (0-9 months)	0.74 ± 1.41	0.598	-0.00 ± 0.00	0.880	0.63 ± 0.42	0.133	0.54 ± 0.78	0.488	0.45 ± 0.56	0.424	0.88 ± 1.23	0.473	-3.25 ± 1.94	0.094	-3.05 ± 1.34	0.023*	-2.37 ± 1.75	0.177
t0-t3 (0-15 months)	0.76 ± 1.29	0.558	0.00 ± 0.00	0.988	0.46 ± 0.43	0.287	0.60 ± 0.71	0.394	0.70 ± 0.72	0.331	1.67 ± 1.19	0.162	-2.67 ± 2.02	0.186	-2.66 ± 1.36	0.051	-2.80 ± 1.47	0.058

Data are presented as regression coefficient ± SE. Statistical significance ($p < 0.05$) is indicated (*). Intervention: n = 104, control: n = 85. ITT: intention-to-treat; BMI: body mass index.

Table S13. Descriptive statistics for blood levels and vascular health (ITT analysis, n = 189).

Group	t0 (0 months)	t1 (3 months)	t2 (9 months)	t3 (15 months)
Fasting plasma glucose (mg/dL)				
Intervention	88.55 ± 9.10	87.38 ± 7.28	86.09 ± 7.40	86.75 ± 6.54
Control	87.71 ± 8.46	87.35 ± 8.82	86.73 ± 9.08	86.56 ± 7.03
HbA_{1c} (%)				
Intervention	5.35 ± 0.34	5.40 ± 0.29	5.42 ± 0.24	5.40 ± 0.24
Control	5.32 ± 0.32	5.32 ± 0.29	5.38 ± 0.28	5.39 ± 0.27
Triglycerides (mg/dL)				
Intervention	114.59 ± 71.68	109.44 ± 56.87	104.70 ± 61.56	100.79 ± 46.85
Control	92.41 ± 32.04	93.29 ± 37.55	93.18 ± 32.23	97.91 ± 41.31
Total cholesterol (mg/dL)				
Intervention	216.30 ± 38.58	215.59 ± 34.92	212.07 ± 31.83	212.14 ± 30.67
Control	217.49 ± 42.04	210.72 ± 37.73	205.63 ± 33.47	209.81 ± 32.06
HDL cholesterol (mg/dL)				
Intervention	63.33 ± 14.21	63.63 ± 12.25	64.04 ± 11.82	64.54 ± 11.92
Control	63.48 ± 13.58	64.27 ± 13.27	62.30 ± 10.99	61.98 ± 11.41
LDL cholesterol (mg/dL)				
Intervention	130.76 ± 30.92	139.19 ± 29.63	140.25 ± 24.63	135.79 ± 24.97
Control	135.48 ± 36.32	136.39 ± 33.31	136.91 ± 27.11	138.08 ± 26.96
FMD (%)				
Intervention	9.04 ± 1.88	9.02 ± 1.74	9.00 ± 1.28	9.54 ± 1.28
Control	9.15 ± 1.92	9.23 ± 2.00	9.29 ± 1.79	9.40 ± 1.53
Vascular age (days)				
Intervention	19,829.27 ± 5946.48	19,873.32 ± 5255.34	19,705.75 ± 4231.25	19,326.56 ± 4052.77
Control	19,556.30 ± 5951.33	18,975.68 ± 5021.05	19,306.42 ± 5080.32	19,583.78 ± 4744.77

Data are presented as mean ± SD. Intervention: n = 104, control: n = 85. ITT: intention-to-treat; HbA_{1c}: glycated hemoglobin; HDL: high-density lipoprotein; LDL: low-density lipoprotein; FMD: flow-mediated dilatation.

Table S14. Effect sizes for blood levels and vascular health (ITT analysis, n = 189).

Group	t0-t1 (0-3 months)	t0-t2 (0-9 months)	t0-t3 (0-15 months)
Fasting plasma glucose (mg/dL)			
Intervention	-0.14 [-0.41, 0.13]	-0.30 [-0.57, -0.02]	-0.23 [-0.50, 0.05]
Control	-0.04 [-0.34, 0.26]	-0.11 [-0.41, 0.19]	-0.15 [-0.45, 0.15]
HbA_{1c} (%)			
Intervention	0.15 [-0.12, 0.43]	0.24 [-0.03, 0.51]	0.17 [-0.10, 0.44]
Control	-0.02 [-0.32, 0.28]	0.21 [-0.10, 0.51]	0.23 [-0.08, 0.53]
Triglycerides (mg/dL)			
Intervention	-0.08 [-0.35, 0.19]	-0.15 [-0.42, 0.12]	-0.23 [-0.50, 0.05]
Control	0.03 [-0.28, 0.33]	0.02 [-0.28, 0.32]	0.15 [-0.15, 0.45]
Total cholesterol (mg/dL)			
Intervention	-0.02 [-0.29, 0.25]	-0.12 [-0.39, 0.15]	-0.12 [-0.39, 0.15]
Control	-0.17 [-0.47, 0.13]	-0.31 [-0.61, -0.01]	-0.21 [-0.51, 0.10]
HDL cholesterol (mg/dL)			
Intervention	0.02 [-0.25, 0.30]	0.06 [-0.22, 0.33]	0.09 [-0.18, 0.36]
Control	0.06 [-0.24, 0.36]	-0.10 [-0.40, 0.22]	-0.12 [-0.42, 0.18]
LDL cholesterol (mg/dL)			
Intervention	0.28 [0.01, 0.55]	0.34 [0.07, 0.61]	0.18 [-0.09, 0.45]
Control	0.03 [-0.28, 0.33]	0.04 [-0.26, 0.35]	0.08 [-0.22, 0.38]
FMD (%)			
Intervention	-0.02 [-0.29, 0.26]	-0.03 [-0.30, 0.25]	0.31 [0.04, 0.59]
Control	0.04 [-0.26, 0.34]	0.08 [-0.23, 0.38]	0.14 [-0.16, 0.44]
Vascular age (days)			
Intervention	0.01 [-0.26, 0.28]	-0.02 [-0.30, 0.25]	-0.10 [-0.37, 0.17]
Control	-0.11 [-0.41, 0.20]	-0.05 [-0.35, 0.26]	0.01 [-0.30, 0.31]

Data are presented as Cohen's d [± 95% CI]. Interpretation: $|d|$ = 0.2: small effect, $|d|$ = 0.5: medium effect, $|d|$ = 0.8: large effect. Intervention: n = 104, control: n = 85. ITT: intention-to-treat; HbA_{1c}: glycated hemoglobin; HDL: high-density lipoprotein; LDL: low-density lipoprotein; FMD: flow-mediated dilatation.

Table S15. Results of robust linear mixed models for blood levels and vascular health (ITT analysis, n = 189).

**Predictors**	**Fasting plasma glucose (mg/dL)**		**HbA_{1c} (%)**		**Triglycerides (mg/dL)**		**Total cholesterol (mg/dL)**		**HDL cholesterol (mg/dL)**		**LDL cholesterol (mg/dL)**		**FMD (%)**		**Vascular age (days)**	
	Estimate	*p*-value	Estimate	*p*-value	Estimate	*p*-value	Estimate	*p*-value	Estimate	*p*-value	Estimate	*p*-value	Estimate	*p*-value	Estimate	*p*-value
(Intercept)	88.43 ± 1.58	< 0.001*	5.40 ± 0.06	< 0.001*	109.84 ± 8.22	< 0.001*	216.19 ± 8.43	< 0.001*	62.76 ± 2.88	< 0.001*	127.30 ± 7.15	< 0.001*	8.89 ± 0.40	< 0.001*	20,944.28 ± 1139.65	< 0.001*
Time																
t0-t1 (0-3 months)	-0.86 ± 1.74	0.622	0.10 ± 0.05	0.077	-4.49 ± 8.20	0.584	2.23 ± 7.77	0.774	-0.19 ± 2.39	0.938	14.60 ± 6.86	0.034*	-0.04 ± 0.58	0.948	520.05 ± 795.35	0.513
t0-t2 (0-9 months)	-3.22 ± 1.81	0.076	0.09 ± 0.06	0.141	-17.39 ± 8.24	0.035*	0.82 ± 7.50	0.913	1.80 ± 3.05	0.556	15.89 ± 7.49	0.035*	0.00 ± 0.60	0.995	193.23 ± 909.50	0.832
t0-t3 (0-15 months)	-2.31 ± 1.79	0.196	0.03 ± 0.05	0.602	-19.37 ± 8.68	0.026*	-3.67 ± 7.41	0.620	2.44 ± 2.78	0.380	7.78 ± 7.98	0.331	0.83 ± 0.61	0.173	-978.62 ± 810.64	0.228
Group (control)	-0.69 ± 1.03	0.507	-0.04 ± 0.04	0.321	-9.43 ± 5.35	0.079	0.07 ± 5.51	0.990	-0.02 ± 1.88	0.992	3.12 ± 4.66	0.502	0.11 ± 0.26	0.661	-557.02 ± 743.91	0.454
Time*group (control)																
t0-t1 (0-3 months)	0.19 ± 1.14	0.864	-0.05 ± 0.04	0.130	1.28 ± 5.36	0.812	-5.07 ± 4.96	0.307	0.23 ± 1.57	0.885	-6.73 ± 4.41	0.128	0.07 ± 0.38	0.858	-654.11 ± 515.92	0.205
t0-t2 (0-9 months)	0.94 ± 1.19	0.433	-0.02 ± 0.04	0.655	7.73 ± 5.36	0.150	-6.21 ± 4.90	0.205	-1.46 ± 1.93	0.448	-6.65 ± 4.82	0.169	0.03 ± 0.40	0.930	-231.30 ± 558.44	0.679
t0-t3 (0-15 months)	0.58 ± 1.24	0.643	0.02 ± 0.04	0.515	10.11 ± 5.61	0.072	-1.66 ± 4.91	0.736	-1.96 ± 1.90	0.301	-2.48 ± 5.13	0.629	-0.33 ± 0.40	0.407	456.45 ± 531.41	0.391

Data are presented as regression coefficient ± SE. Statistical significance ($p < 0.05$) is indicated (*). Intervention: n = 104, control: n = 85. ITT: intention-to-treat; HbA_{1c}: glycated hemoglobin; HDL: high-density lipoprotein; LDL: low-density lipoprotein; FMD: flow-mediated dilatation.
